# Herd immunity and prevention in HPV transmission with exogenous reinfection

**DOI:** 10.1371/journal.pone.0327233

**Published:** 2025-07-11

**Authors:** Md Mehedi Hasan, Md Hamidul Islam

**Affiliations:** Department of Applied Mathematics, University of Rajshahi, Rajshahi, Bangladesh; Shanxi University, CHINA

## Abstract

Cervical cancer is a major global health concern, primarily caused by human papillomavirus (HPV), which spreads through unsafe sexual contact. Natural immunity often fails to provide full protection, permitting exogenous reinfection, which plays a key role in the persistence and transmission of HPV. This paper presents and analyzes a deterministic mathematical model of HPV transmission, incorporating exogenous re-infection. We utilized VIA test result data from 2019 to 2023 in Bangladesh to estimate key parameter values. The findings were validated through numerical simulations using MATLAB, and the analytical results demonstrated strong consistency with the numerical outputs. A critical insight is the presence of reinfection-induced backward bifurcation, indicating that targeting primary infections alone is insufficient for eradication, highlighting the need to address exogenous reinfection for effective control. We propose optimal control strategies tailored to resource-limited settings like Bangladesh, where limited vaccine access presents significant challenges, and community-driven education on safe sexual practices is crucial and continuously evolving. The results emphasize that the most effective approach combines targeted prevention with widespread community education, while precision in intervention implementation is essential to prevent reinfection surges that could prolong disease persistence and undermine control efforts. These insights provide a strong foundation for refining intervention strategies and offer valuable guidance for controlling HPV transmission through non-pharmaceutical interventions, particularly in resource-constrained settings.

## 1 Introduction

Human papillomavirus (HPV) is a non-enveloped, double-stranded DNA virus that can infect individuals of all sexes and is regarded as one of the most common sexually transmitted infections worldwide [1,2]. HPV is primarily transmitted through sexual contact and is not spread through blood. There are over 200 known types of HPV, with more than 40 capable of infecting the epithelial tissues in the anogenital region [2,3]. HPV is classified into high-risk and low-risk types based on the severity of infection in the anogenital area. Low-risk HPV strains typically lead to genital warts, which often resolve on their own due to natural immunity. In contrast, high-risk strains, such as HPV 16 and HPV 18, are associated with an increased risk of cervical cancer [4]. Approximately 70% of cervical cancer cases in women are associated with HPV strains 16 and 18 [5].

People in polygamous relationships are at an increased risk of developing an HPV infection during their lifetime. In most cases, HPV does not exhibit any clinical signs in the early stages. Due to its asymptomatic nature, the virus can spread within the population without any noticeable symptoms. While most HPV infections typically resolve within two years, persistent infections with oncogenic HPV types can lead to cervical cancer if not cleared by the immune system [6]. Cervical cancer is one of the most commonly diagnosed gynecologic cancers in women worldwide, despite being preventable through early detection. It ranks fourth among all cancers affecting women, with over 600,000 new cases and 341,831 deaths reported in 2020 [7].

Cervical cancer is the second most common cancer in Bangladesh and ranks third for cancer-related deaths among women [8]. Mortality rates in Bangladesh are high due to inadequate treatment, delayed detection, insufficient screening programs, and poor socioeconomic conditions. Approximately 8,268 new cases of cervical cancer are diagnosed each year, with around 4,971 deaths attributed to the disease annually (2020 estimates) [8]. It is the second most common type of cancer among Bangladeshi women between the ages of 15 and 44 [8]. Approximately 7.9% of women are affected by cervical cancer, with around 82.8% of these cases linked to HPV strains 16 or 18[9].

A number of mathematical models have been developed to gain insights into the transmission dynamics of HPV and to evaluate the effectiveness of different interventions. Praveen *et al*. [10] proposed a mathematical model to examine HPV transmission and cervical cancer in India, concluding that effective disease control relies on lowering transmission rates. Lee *et al*. [11] developed a mathematical model to evaluate HPV prevention and mitigation strategies in the United States. Mumtaz *et al*. [12] proposed a model for HPV transmission and cervical cancer prevention, highlighting that the most effective approach to controlling HPV and preventing cancer is to implement multiple vaccines with high coverage. Malik *et al*. [13] created a two-sex mathematical model to assess the impact of vaccination and Pap screening on HPV transmission dynamics. One of their key findings is that the model demonstrates backward bifurcation due to imperfect vaccination and disease-induced mortality.

Gao *et al*. [14] developed a two-sex mathematical model to analyze the transmission of HPV and evaluate different vaccination strategies. The model, which divides heterosexually active males and females into susceptible, vaccinated, and infected groups, explores optimal vaccine allocation to minimize disease prevalence. One key finding is that prioritizing vaccination for the gender with a lower recruitment rate, combined with adjusting vaccine distribution over time, is more effective than equal distribution. Several other models related to HPV and cervical cancer have specifically addressed the impact of control measures, including prevention, screening, vaccination, and treatment [15–19].

Exogenous re-infection refers to the process of acquiring an HPV infection again from external sources, such as sexual contact, after having previously recovered from the virus. This occurs when an individual has either recovered from an initial infection or developed some immunity to a specific strain of HPV and is subsequently re-exposed to the virus, whether from the same strain or a different one. Ranjeva *et al*. [20] found that an initial HPV infection increases the likelihood of contracting the same type of virus again, rather than providing protective immunity. Helen Trottier *et al*. [21] investigated the link between HPV infection or reinfection and having a new sexual partner, concluding that natural immunity does not reduce the risk of reinfections. Their study indicated that both initial infections and reinfections, whether from the same or different HPV types, were strongly associated with the presence of new sexual partners. Additionally, Wilson *et al*. [22] demonstrated that naturally acquired antibodies do not fully protect women from reinfection. While several mathematical studies have explored the impact of exogenous reinfection in other diseases, such as tuberculosis [23–25], Chlamydia trachomatis [26], and HPV-syphilis [27], the effect of exogenous reinfection on HPV dynamics has not been extensively studied to date.

Considering the biological evidence of exogenous reinfection in HPV dynamics, we formulated a mathematical model to analyze HPV transmission dynamics and assess the impact of exogenous reinfection. The primary objective of this model is to evaluate the effects of external reinfection on cervical cancer incidence and overall transmission dynamics. We began with a comprehensive mathematical analysis of the model, followed by numerical simulations to validate our theoretical conclusions. To ensure the model’s accuracy, we utilized the maximum likelihood estimation approach, drawing on data from the National Cervical and Breast Cancer Statistics of Bangladesh, specifically from the VIA (Visual Inspection with Acetic Acid) test results collected between 2019 and 2023.

The remainder of this paper is structured as follows: Sect 2 outlines the proposed model and details its mathematical analysis. In Sect 3, we explore the techniques used for parameter estimation, present the results with a thorough discussion of their implications, and provide numerical validation of backward bifurcation. In Sect 4, we examine the efforts required to control and contain transmission. Building on the insights gained in this section, we propose an optimal control model in Sect 5. Finally, Sect 6 concludes the paper, summarizing the main findings.

## 2 Mathematical model

### 2.1 Model formulation

In this section, we present a compartmental model that illustrates the dynamics of HPV transmission and the subsequent emergence of cervical cancer within the population of Bangladesh, including the potential for exogenous reinfection of HPV. To achieve this, we extend the standard SEIR compartmental model [28,29] by incorporating a cervical cancer compartment (C), forming the SEICR (Susceptible-Exposed-Infected-Cervical Cancer-Recovered) framework.

**Fig 1:**
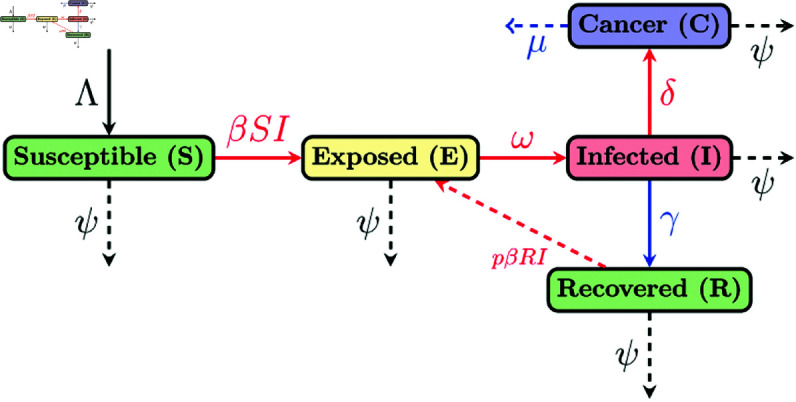
Schematic diagram. A schematic representation illustrates the flow between compartments, with solid red lines indicating transitions from Susceptible to Exposed, Exposed to Infected, and Infected to Cancer, and a red dashed line from Recovered back to Exposed. The solid blue arrow represents the pathway from Infected to Recovery. Dashed arrows denote death rates (ψ) from each compartment, while the downward arrow above Susceptible indicates the birth rate (Λ). The dashed blue arrow represents the disease-induced death due to cervical cancer.

The total population, *N*(*t*), is divided into five distinct compartments: Susceptible individuals *S*(*t*), HPV-exposed individuals *E*(*t*), HPV-infected individuals *I*(*t*), Recovered individuals *R*(*t*), Cervical cancer individuals *C*(*t*). As HPV is a sexually transmitted infection, transmission occurs exclusively when susceptible *S*(*t*) and infected *I*(*t*) individuals engage in sexual contact. The schematic diagram presented in this study illustrates the dynamics of the model (see Fig 1). Λ represents the rate at which new individuals enter the population and are initially classified as susceptible to HPV infection. At a transmission rate of β, susceptible individuals interact with HPV-infected people, which leads to their progression to the exposed compartment *E*(*t*). This interaction signifies the initial step in the transmission dynamics of the virus. We assume ω is the transition rate from the exposed compartment *E*(*t*) to the infectious compartment *I*(*t*). Assume that people with HPV develop cervical cancer at a rate δ. A portion of the infected population will recover from HPV due to the immune system at the rate γ, transitioning to the recovered compartment *R*(*t*). Recovered individuals can be re-infected at a rate of pβRI, with *p* indicating the factor that elevates the re-infection rate. In the model, ψ represents the natural death rate affecting individuals in every compartment, while μ denotes the disease-induced mortality rate specifically associated with cervical cancer. In our model, we assume that individuals with cervical cancer are critically ill and not responsible for new infections, as they are assumed not to engage in unsafe sexual activity.

Considering the above description, we formulate the following system of nonlinear ordinary differential equations (ODEs):


$$\frac{dS}{dt}=\Lambda -\beta IS-\psi S$$

$$\frac{dE}{dt}=\beta IS+p\beta RI-\psi E-\omega E$$

$$\frac{dI}{dt}=\omega E-\gamma I-\delta I-\psi I$$

$$\frac{dR}{dt}=\gamma I-p\beta RI-\psi R$$

$$\frac{dC}{dt}=\delta I-\mu C-\psi C$$ (1)

with initial conditions *S*(0) > 0, E(0)≥0, I(0)≥0, C(0)≥0. Table 1 provides a summary of the model parameters along with their definitions.

**Table 1:** Model parameters and their meaning.

Parameter	Description
Λ	Birth rate of susceptible
ψ	Natural death rate
β	HPV transmission rate
γ	Recovery rate from HPV
ω	Progression rate from exposed to HPV infectious chamber
δ	Progression rate of Cervical cancer from HPV
μ	Cervical cancer-induced death rate
*p*	Factor boosting re-infection rate

### 2.2 Mathematical analysis

#### 2.2.1 Positivity of solution.

**Theorem 1.** *The solutions to the system remain non-negative subjected to the non-negative initial conditions at t > 0.*

*Proof:* We begin by rewriting the first equation from (1) as follows:

$$\frac{dS}{dt}=\Lambda -\beta IS-\psi S\;\frac{dS}{dt}+(\beta I+\psi )S=\Lambda$$ (2)

Multiplying both sides by $e^{\int (\beta I+\psi )\;dt}$ we get,


$$\frac{d}{dt}\left(Se^{\int (\beta I+\psi )\;dt}\right)=\Lambda e^{\int (\beta I+\psi )\;dt}$$

By solving the equation, we get

$$S(t)=S(0)e^{-\int_{0}^{t}(\beta I+\psi )\;dt}+e^{-\int_{0}^{t}(\beta I+\psi )\;dt}\int_{0}^{t}\Lambda e^{\int_{0}^{z}(\beta I+\psi )\;dz}\;dz$$ (3)

Using a similar approach, we obtain


$$E(t)=E(0)e^{-\int_{0}^{t}(\omega +\psi )\;dt}+e^{-\int_{0}^{t}(\omega +\psi )\;dt}\int_{0}^{t}(\beta SI+p\beta RI)e^{\int_{0}^{z}(\omega +\psi )\;dz}\;dz$$

$$I(t)=I(0)e^{-\int_{0}^{t}(\gamma +\delta +\psi )\;dt}+e^{-\int_{0}^{t}(\gamma +\delta +\psi )\;dt}\int_{0}^{t}(\omega E)e^{\int_{0}^{z}(\gamma +\delta +\psi )\;dz}\;dz$$

$$R(t)=R(0)e^{-\int_{0}^{t}(p\beta I+\psi )\;dt}+e^{-\int_{0}^{t}(p\beta I+\psi )\;dt}\int_{0}^{t}(\gamma I)e^{\int_{0}^{z}(p\beta I+\psi )\;dz}\;dz$$

$$C(t)=C(0)e^{-\int_{0}^{t}(\mu +\psi )\;dt}+e^{-\int_{0}^{t}(\mu +\psi )\;dt}\int_{0}^{t}(\delta I)e^{\int_{0}^{z}(\mu +\psi )\;dz}\;dz$$

A negative exponential function is always greater than or equal to zero, and since *S*(0) > 0, it follows that *S*(*t*) > 0 for *t* > 0. Similarly, E(t)≥0, I(t)≥0, R(t)≥0, and C(t)≥0 for all *t* > 0. This completes the proof. ◻

#### 2.2.2 Invariant region.

The relation N(t)=S(t)+E(t)+I(t)+R(t)+C(t) represents the total population. The following expression can be obtained by summing the differential equations for each compartment in system (1):

$$\frac{dN}{dt}=\Lambda -\psi S-\psi E-\psi I-\psi R-\psi C-\mu C\;\frac{dN}{dt}\leq \Lambda -N\psi$$ (4)

Solving the above equation we get,


$$\Omega \;=\{S,E,I,R,C\in {\mathbb{R}}_{5}^{+}:0\leq N(t)\leq \frac{\Lambda }{\psi }\}$$

Here, the set Ω is positively invariant, implying that the solutions to the system (1) remain bounded within this region.

#### 2.2.3 Basic reproduction number.

The disease-free equilibrium (DFE) point of the system is $E_{0}=\left(\frac{\Lambda }{\psi },0,0,0,0\right)$. Next, we apply the next-generation matrix method [30] to calculate the basic reproduction number (*R*_0_), which is determined as follows:


$$R_{0}=\rho (F_{0}V_{0}^{-1})=\frac{\beta \Lambda \omega }{\psi (\omega +\psi )(\gamma +\delta +\psi )}$$

where

$$F_{0}=[\begin{array}{cc} 0 & \frac{\beta \Lambda }{\psi }\\ 0 & 0 \end{array}]\;\text{and}\;V_{0}=[\begin{array}{cc} \left(\omega +\psi \right) & 0\\ -\omega & \left(\gamma +\delta +\psi \right) \end{array}].$$ (5)

The expression of *R*_0_ can be reorganized as

$$R_{0}=\frac{1}{\gamma +\delta +\psi }\times \frac{\beta \Lambda }{\psi }\times \frac{\omega }{\omega +\psi }\;=\text{average infectious lifetime}\times \text{number of susceptible receiving the virus}\;\times \text{the probability of developing infections}.$$ (6)

The expression for *R*_0_ is therefore well-defined.

#### 2.2.4 Local stability at disease-free equilibrium.

**Theorem 2.** *The disease-free equilibrium *E*_*0*_ is locally asymptotically stable when *R*_*0*_*<1, otherwise it is unstable.**

*Proof:* The Jacobian matrix J evaluated at the *E*_0_ is expressed as:

$$J_{0}=[\begin{array}{ccccc} -\psi & 0 & -\frac{\beta \Lambda }{\psi } & 0 & 0\\ 0 & -(\omega +\psi ) & \frac{\beta \Lambda }{\psi } & 0 & 0\\ 0 & \omega & -(\gamma +\delta +\psi ) & 0 & 0\\ 0 & 0 & \gamma & -\psi & 0\\ 0 & 0 & \delta & 0 & -(\psi +\mu ) \end{array}]$$ (7)

We obtain three eigenvalues $\lambda_{1}=-\psi$ and $\lambda_{2}=-(\psi +\mu )$, $\lambda_{3}=-\psi$. Solving the following characteristic equation yields the other two eigenvalues:

$$\lambda^{2}+A\lambda +B(1-R_{0})=0$$ (8)

where A=(δ+ψ+ω+γ+ψ) and B=(ω+ψ)(γ+δ+ψ). The other two eigenvalues are negative when *R*_0_<1, which indicates that the disease-free equilibrium *E*_0_ is locally asymptotically stable. When *R*_0_<1, the Jacobian has negative eigenvalues. In contrast, from (8), we noticed that when *R*_0_>1, there is a positive eigenvalue of the Jacobian matrix *J*_0_, ensuring the instability of the disease-free equilibrium (DFE). Hence, the DFE is stable when *R*_0_<1; otherwise, it is unstable for *R*_0_>1. ◻

The global stability of the disease-free equilibrium is also verified by using the Lyapunov function. The details of the analysis are shown in S1 Appendix.

#### 2.2.5 Existence and stability of endemic equilibrium.

Endemic equilibrium (EE) occurs when the infection persists within the population. Calculating this condition is crucial for understanding the disease’s persistence and spread. Setting the right-hand side of the system in the model (1) to zero yields the solutions for the endemic equilibrium, resulting in the following outcomes:


$$S^{*}=\frac{\Lambda }{\beta I^{*}+\psi },\;E^{*}=\frac{(\gamma +\delta +\psi )I^{*}}{\omega },\;R^{*}=\frac{\gamma I^{*}}{p\beta I^{*}+\psi },\;C^{*}=\frac{\delta I^{*}}{\left(\psi +\mu \right)}$$

and,

$$AI^{*2}+BI^{*}+C=0,$$ (9)

where,

$$A=-p\beta^{2}(\omega \delta +\omega \psi +\psi \delta +\psi \gamma +\psi^{2})\;B=\omega p\beta^{2}\Lambda +\omega p\beta \gamma \psi -\psi (\omega +\psi )(\gamma +\delta +\psi )(1+p)\;C=\psi^{2}(\omega +\psi )(\gamma +\delta +\psi )(R_{0}-1)$$ (10)

From Eq (10) we observe that the value of *A* is always negative. The signs of the coefficients *B* and *C* dictate the number of positive real roots the polynomial may have, as determined by Descartes’ Rule of Signs. Consequently, we obtain the following result:

**Theorem 3.** *The system (*1*) has*

i) *exactly one unique endemic equilibrium if *R*_*0*_*>1.**
ii) *exactly two endemic equilibria if B > 0, C<0, and ${\text{B}}^{2}$–4AC > 0.*

However, *C* is negative only when *R*_0_<1. The presence of multiple endemic equilibria when *R*_0_<1 indicates the potential for backward bifurcation. This context suggests that the coexistence of a stable disease-free equilibrium with a stable endemic equilibrium is possible even when *R*_0_ is below one. To identify this bi-stable region, we need to calculate another threshold quantity that delineates the coexistence area, known as the backward bifurcation threshold *R*_*c*_ which is


$$R_{c}=\frac{\sqrt{b^{2}-4ac}-b}{2p^{2}\omega \Lambda \psi (\omega +\psi )(\gamma +\delta +\psi )}.$$

The detailed derivation of *R*_*c*_ and its physical significance is outlined in S2 Appendix. Hence, we get the following theorem:

**Theorem 4.** *If $R_{c}<R_{0}<1$ and B > 0 then there exist two distinct endemic equilibria. Backward bifurcation occurs within the interval $R_{c}<R_{0}<1$.*

The presence of backward bifurcation is analyzed utilizing the Center Manifold Theory, as extensively developed by Castillo-Chavez and Song [31]. Before applying this theory, some simplification and variable adjustments are required. We set *x*_1_ = *S*, *x*_2_ = *E*, *x*_3_ = *I*, *x*_4_ = *R*. In vector notation, we represent these variables as $x={\left(x_{1},x_{2},x_{3},x_{4}\right)}^{T}$.


$$\frac{dx_{1}}{dt}=f_{1}(x)=\Lambda -\beta x_{1}x_{3}-\psi x_{1}$$

$$\frac{dx_{2}}{dt}=f_{2}(x)=\beta x_{1}x_{3}+p\beta x_{4}x_{3}-\psi x_{2}-\omega x_{2}$$

$$\frac{dx_{3}}{dt}=f_{3}(x)=\omega x_{2}-\gamma x_{3}-\delta x_{3}-\psi x_{3}$$

$$\frac{dx_{4}}{dt}=f_{4}(x)=\gamma x_{3}-p\beta x_{4}x_{3}-\psi x_{4}$$ (11)

Now, consider the case where *R*_0_ = 1 and $\beta =\beta^{*}$ is chosen as the bifurcation parameter which is defined by:


$$\beta =\beta^{*}=\frac{\psi (\omega +\psi )(\gamma +\delta +\psi )}{\Lambda \omega }$$

Now we evaluate the Jacobian of the model at the disease-free equilibrium (DFE) as follows:

$$J_{\left(E_{0},\beta^{*}\right)}=[\begin{array}{cccc} -\psi & 0 & -\frac{\beta^{*}\Lambda }{\psi } & 0\\ 0 & -(\omega +\psi ) & \frac{\beta^{*}\Lambda }{\psi } & 0\\ 0 & \omega & -(\gamma +\delta +\psi ) & 0\\ 0 & 0 & \gamma & -\psi \end{array}]$$ (12)

The components of the right eigenvectors are derived by solving the equation *Ju* = 0, where $u={\left(u_{1},u_{2},u_{3},u_{4}\right)}^{T}$. The resulting values of the right eigenvectors are as follows:


$$u_{1}=-\frac{1}{\psi }\left[\frac{\left(\omega +\psi )(\gamma +\delta +\psi \right)}{\omega }\right]u_{3},\;u_{2}=\frac{\left(\gamma +\delta +\psi \right)}{\omega }u_{3},\;u_{3}=u_{3},\;u_{4}=\frac{\gamma }{\psi }u_{3}$$

To compute left eigenvector solving the equation $vJ_{(E_{0},\beta^{)}}=0$ where $v=(v_{1},v_{2},v_{3},v_{4})$. We obtained the value of the left eigenvector,


$$v_{1}=v_{4}=0,\;v_{2}=\frac{\omega }{\left(\omega +\psi \right)}v_{3}$$

Hence the value of bifurcation coefficients is,

$$a=\sum_{k,i,j=1}^{4}v_{k}u_{i}u_{j}\frac{\partial^{2}f_{k}}{\partial x_{i}\partial x_{j}}(E^{0},\beta^{*})\;=\frac{2v_{2}u_{3}^{2}\beta^{*}\gamma }{\psi }[p-\frac{\left(\omega +\psi )(\gamma +\delta +\psi \right)}{\omega \gamma }]$$ (13)

$$b=\sum_{k,i=1}^{4}v_{k}u_{i}\frac{\partial^{2}f_{k}}{\partial x_{i}\partial \beta }(E^{0},\beta^{*})\;=v_{2}u_{3}\frac{\Lambda }{\psi }$$ (14)

The conditions of backward bifurcation outlined in [31] is that *a* > 0 and *b* > 0. From Eq (14), it is evident that *b* is always positive, i.e.,*b* > 0. Additionally, based on Eq (13), *a* is positive if and only if the following condition is satisfied:


$$p>\frac{\left(\omega +\psi )(\gamma +\delta +\psi \right)}{\omega \gamma }$$

Therefore, we can conclude that if $p>\frac{\left(\omega +\psi )(\gamma +\delta +\psi \right)}{\omega \gamma }$, then system (1) undergoes a backward bifurcation at *R*_0_ = 1. Hence the following theorem:

**Theorem 5.** *If $p>\frac{\left(\omega +\psi )(\gamma +\delta +\psi \right)}{\omega \gamma }$, then the system (1) undergoes a backward bifurcation at R_0_ = 1.*

Rearranging the inequality $p>\frac{\left(\omega +\psi )(\gamma +\delta +\psi \right)}{\omega \gamma }$ results in *R*_*p*_>1, where

$$R_{p}=\frac{p\omega \gamma }{\left(\omega +\psi )(\gamma +\delta +\psi \right)}\;=\frac{\gamma }{\left(\gamma +\delta +\psi \right)}\times p\times \frac{\omega }{\left(\omega +\psi \right)}\;=\text{probability of recovery}\times \text{factor boosting re-infection rate}\;\times \text{probability of developing infection}.$$ (15)

We refer to *R*_*p*_ as the basic re-infection number, as it represents the average number of re-infections from previously recovered individuals. The analysis above suggests that when *R*_*p*_>1, the system (1) may exhibit backward bifurcation. Conversely, when *R*_*p*_<1, the likelihood of backward bifurcation diminishes, and only forward bifurcation occurs.

**Fig 2:**
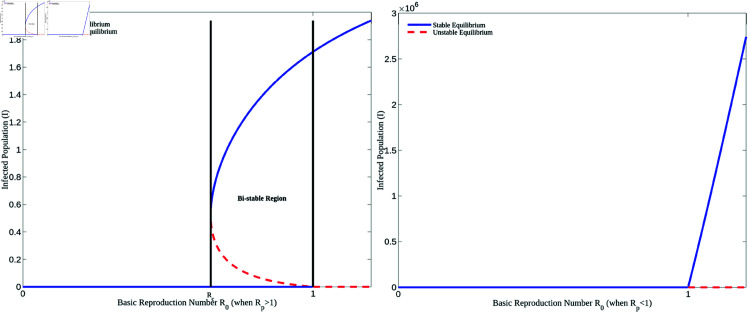
Backward and forward bifurcation. Figures illustrating the backward and forward bifurcation of the HPV model (1) according as *R*_*p*_>1 and *R*_*p*_<1, respectively.

Fig 2 displays the bifurcation diagrams of the HPV transmission model. In the left panel, the model demonstrates backward bifurcation with the following parameter values: Λ=2220800, *p* = 3.5, ω=0.6681, γ=0.5, ψ=0.01388, μ=0.63, δ=0.05. This scenario arises when the basic re-infection number exceeds 1. In contrast, as shown in the right panel, when *R*_*p*_<1, the model exhibits forward bifurcation.

## 3 Numerical simulations

### 3.1 Initial conditions

The initial conditions for our model were determined using available epidemiological and demographic data from Bangladesh. Since official data on HPV-positive cases are unavailable, we used VIA test results from 2019 to 2023 as a proxy for HPV infection.

In 2019, the reported number of VIA-positive cases was 7,502, which we considered as the initial infected population, *I*(0). Since direct epidemiological data on the exposed population are unavailable, estimating *E*(0) presents a challenge. For infections like HPV, exposed individuals are typically asymptomatic and not systematically tracked, making it difficult to obtain reliable estimates of exposure levels. Given this limitation, we selected E(0)=4000, which is approximately half of *I*(0). This choice ensures that the model accurately captures the observed trends while maintaining biological plausibility.

In 2019, the reported number of cervical cancer cases was 8,068 [32], which we considered as the initial cancer population, *C*(0). The initial recovered population was assumed to be zero at the start of our simulation, i.e., R(0)=0. The total population was obtained from demographic data and estimated as $N(0)=160\times 10^{6}$. Therefore, the initial susceptible population was then computed as S(0)=N(0)−E(0)−I(0)−R(0)−C(0).

Thus, the initial conditions applied in our model are: $N(0)=160\times 10^{6}$, E(0)=4000, I(0)=7502, R(0)=0, C(0)=8068, S(0)=N(0)−E(0)−I(0)−R(0)−C(0).

These initial values were used consistently across all simulations in this study unless stated otherwise.

### 3.2 Parameter estimation

The system in Eq (1) includes seven parameters, some of which are derived from both demographic data specific to the population of Bangladesh and pathophysiological information related to HPV. For instance, the life expectancy of the population in Bangladesh is estimated to be 72 years [33], allowing the natural death rate, ψ, to be calculated as $\psi =\frac{1}{72}$ per year. Given a total population of around 160 million [33], the recruitment rate Λ— representing the annual influx of new individuals into the susceptible population— is calculated using the formula Λ=ψN, resulting in Λ=2,220,800 per year. HPV infections generally resolve within two years without exhibiting any symptoms [6], and we assume a recovery rate of γ=0.5 per year, as also considered by other authors [10,34]. The parameter δ indicates the rate at which individuals with persistent HPV infections progress to cervical cancer. Since cervical cancer can develop 15 to 20 years after an HPV infection [35], we assume the progression rate δ to be $\frac{1}{20}=0.05$ per year. The disease-induced death rate was calculated by taking the ratio of average annual cancer-related deaths to the average number of new cases in Bangladesh [32], resulting in μ=0.63.

The remaining model parameters were estimated using the Maximum Likelihood fitting method [36], utilizing data from Bangladesh’s national cervical cancer screening program, which employs Visual Inspection with Acetic Acid (VIA) [37]. The dataset, spanning 2019 to 2023 (see S1 Table), was extracted in an aggregated format from the DHIS2-based electronic repository using MS Excel. It includes screening data from a broad spectrum of healthcare facilities, ranging from primary and secondary care centers to advanced medical institutions and specialized hospitals dedicated to cancer treatment. Since official records of HPV-confirmed cases are unavailable in Bangladesh, the model was calibrated using anonymized annual data on VIA-positive cases extracted from this repository.

### 3.3 Identifiability and data fitting

Let X=(S,E,I,R,C) represent the system’s state variables, and let the right-hand side of the system in Eq (1) be denoted by 𝔽. We define ℙ=(β,ω) as the vector of parameters to be estimated, while the remaining parameters, which are known, are outlined in Table 2 with the appropriate citation. Here, I(t,ℙ) refers to the vector of expected values, and $\hat{I}(t,\mathbb{P})$ represents the observed data at time points t=1,2,…,5 years. We assume that $\hat{I}(t,\mathbb{P})$ follows a Poisson distribution with a mean of I(t,ℙ). Thus, the probability mass function (PMF) of the Poisson distribution is given by:

**Table 2:** The parameter values used in the numerical calculations.

Parameter	Value (year)^−1^	Source
Λ	2220800	[33]
β	$9.0026\times 10^{-09}$	Fitted
ψ	0.013888	[33]
ω	0.6681	Fitted
δ	0.05	[35]
γ	0.5	[10]
μ	0.63	[32]
*p*	>1	Assumed


$$P(\hat{I}(t,\mathbb{P})=k)=\frac{I(t,\mathbb{P}{)}^{k}e^{-I(t,\mathbb{P})}}{k!},\;k=1,2,\ldots$$

The maximum likelihood function is obtained by taking the product of the individual Poisson PMFs for the observed data $\hat{I}(t,\mathbb{P})$ over t=1,2,…,5 years, as follows:


$$L(\mathbb{P})=\prod_{t=1}^{5}\frac{I(t,\mathbb{P}{)}^{\hat{I}(t,\mathbb{P})}e^{-I(t,\mathbb{P})}}{\hat{I}(t,\mathbb{P})!}$$

The Log-Likelihood function is obtained by taking the natural logarithm of the likelihood function described above. Our objective is to estimate the parameters ℙ=(β,ω) that maximize this Log-Likelihood. However, since the logarithmic function is monotonically increasing, it is computationally more convenient to minimize the Negative Log-Likelihood function instead of maximizing the Log-Likelihood itself. The Negative Log-Likelihood function is given by


$$N\ell (\mathbb{P})=\sum_{t=1}^{5}\left[-\hat{I}(t,\mathbb{P})\log(I(t,\mathbb{P}))+I(t,\mathbb{P})+\log(\hat{I}(t,\mathbb{P})!)\right]$$

Since the last term in the above equation remains constant, it is enough to focus on minimizing the sum of the first two terms. As a result, the fitting procedure simplifies to a minimization problem given by:


$$min\left(N\ell (\mathbb{P})\right)=min\left(\sum_{t=1}^{5}\left[-\hat{I}(t,\mathbb{P})\log(I(t,\mathbb{P}))+I(t,\mathbb{P})\right]\right)$$

subject to

$$\frac{d}{dt}X(t,\mathbb{P})=𝔽(X,\mathbb{P},t)\;I(0)=I_{0}\;X(t),\mathbb{P}\geq 0$$ (16)

In this study, the technique for testing identifiability is based on methods outlined in [36]. The parameters ℙ are considered identifiable if a unique solution exists for the system X(t,ℙ) with a fixed initial condition. This involves both *structural* and *practical identifiability* tests. First, we estimate the *Fisher Information Matrix (FIM)*, which quantifies the sensitivity of the model output to changes in parameters. To confirm the structural identifiability, we check if the rank of the FIM is full, ensuring no implicit dependencies between the parameters.

Our system is characterized by five observations, five state variables, and two unknown parameters. For the minimization problem in (16), the Fisher Information Matrix (FIM) is of order 2×2 and is defined as $\text{FIM}=M^{T}M$, where *M* is the sensitivity matrix. We numerically compute the FIM and check its rank, which is 2, thereby ensuring the *structural* identifiability of the parameters. The detailed derivation of the sensitivity matrix *M* is provided in [36].

For practical identifiability, we compute *profile likelihoods* for each parameter. These likelihoods indicate how the Negative Log-Likelihood Nℓ(ℙ) depends on each parameter and reveal the location of the minima at the estimated parameter values. The profile likelihoods are computed by fixing one parameter and minimizing the Nℓ(ℙ) with respect to the other, allowing us to investigate the distinct contributions of each parameter within the model.

**Fig 3:**
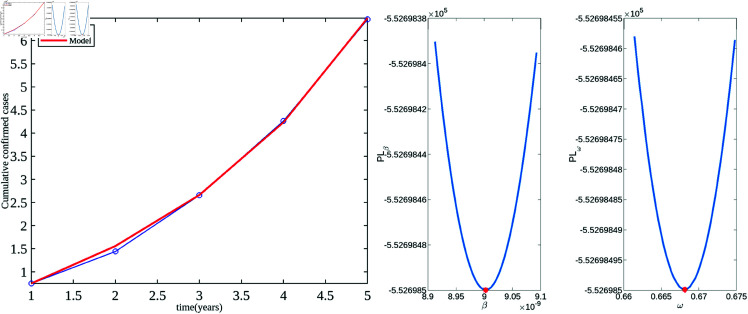
Data fitting and comparison. Graphs displaying the estimated parameters β and ω, with data fitting on the left and their profile likelihoods on the right.

Fig 3 presents the data fitting for the HPV model along with the profile likelihoods of the estimated parameters. The profile likelihoods distinctly show the unique minima of Nℓ(ℙ) at the estimated parameter values, thereby confirming the structural identifiability of the parameters.

According to our estimated results, the transmission rate is $\beta =9.0026\times 10^{-9}$, and the progression rate from the exposed to the HPV-infectious stage is ω=0.6681. Based on the value of ω, the estimated latent period is approximately 1.496 years, which is reasonable and closely aligns with values reported in the literature. For instance, Zhang *et al*. and Alsaleh *et al*. [38,39] used a progression rate of 0.5 for the transition from the exposed stage to the infectious stage.

Table 2 provides a summary of the parameter values.

### 3.4 Basic results

In this subsection, we provide key numerical results for the model (1) that reinforce the findings from the earlier mathematical analyses. The simulations were carried out using MATLAB’s ODE45 solver, with the parameter values listed in Table 2.

**Fig 4:**
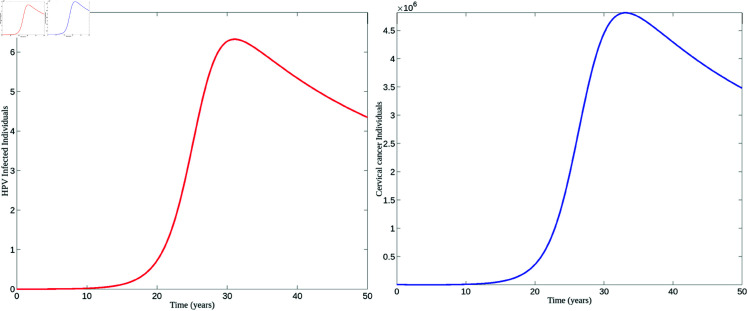
Disease persistence. Graphs depicting disease dynamics for *R*_0_>1. The left figure represents the population of HPV-infected individuals, while the right figure illustrates the population of individuals with cervical cancer.

Fig 4 demonstrates the disease dynamics when *R*_0_>1. Based on the parameter values presented in Table 2, *R*_0_ = 2.50. Since *R*_0_>1, the infection spreads rapidly, leading to a significant rise in the number of infected individuals. This figure also shows that the number of cervical cancer cases increases alongside the number of infected individuals. The stabilization observed suggests the continued presence of cervical cancer in the population as a result of ongoing HPV infections.

**Fig 5:**
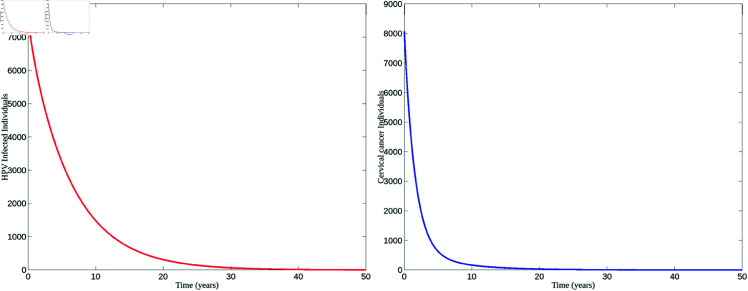
Disease extinction. Graphs depicting disease dynamics for *R*_0_<1, similar to the graphs shown in Fig 4.

Conversely, the disease is observed to die out when *R*_0_<1. The results for this scenario are shown in Fig 5. As seen in Fig 5, the number of HPV-infected individuals declines, and over time, the disease is eradicated from the population. Similarly, cervical cancer cases decrease alongside the reduction in infected individuals.

### 3.5 Validation of backward bifurcation

Based on our theoretical analysis outlined in Theorem 5, backward bifurcation occurs in the interval $R_{c}<R_{0}<1$, where *R*_*c*_ represents the backward bifurcation threshold. In this context, the region defined by $R_{c}<R_{0}<1$ is a bi-stable area where both stable DFE and endemic equilibria coexist.

**Fig 6:**
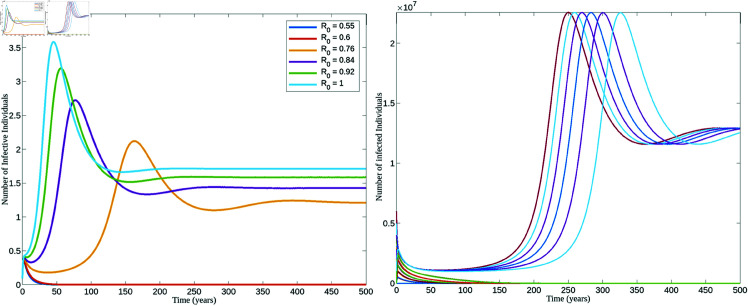
Endemicity and disease extinction based on *R*_0_ and initial infected and exposed populations. Figures illustrating the convergence to DFE or EEP for different values of *R*_0_ and varying sizes of the initial infected population: for $R_{0}\in (0.5,1)$ with *R*_*c*_ = 0.64 (left), and for different initial conditions (right).

Fig 6 illustrates how the system converges to either the DFE or the EEP, being influenced by the fixed initial conditions and various parameter values that satisfy $R_{0}\in [R_{c},1]$. This figure also illustrates how the system converges to either the DFE or the EEP based on different values of *I*(0). The following parameter values were applied: Λ=2220800, *p* = 3.5, ω = 0.6681, γ=0.5, and β was defined in terms of *R*_0_. The remaining parameters were applied as specified in Table 2. Using these values, the critical threshold is calculated to be *R*_*c*_ = 0.64. The parameter β was calculated for each *R*_0_ within the range $R_{0}\in [0.5,1]$. This interval was chosen primarily to ensure that some β values fall outside the backward bifurcation range. The graph shown in the left of this figure clearly confirms the existence of backward bifurcation in the model, as for the same initial condition but different values of *R*_0_ that falls within and outside the backward bifurcation range leads to the two different outcomes.

This figure also illustrates that even when *R*_0_<1 but it falls within the range $R_{c}<R_{0}<1$, both a disease-free equilibrium (DFE) and an endemic equilibrium (EEP) can coexist depending on the initial infection. The disease tends to die out for small initial infective populations with limited exposures, driving the system toward a Disease-Free Equilibrium (DFE). However, with a larger initial infection, the system may become overwhelmed, leading to the disease stabilizing at an endemic state.

#### 3.5.1 Basin of attraction for DFE and EEP.

**Fig 7:**
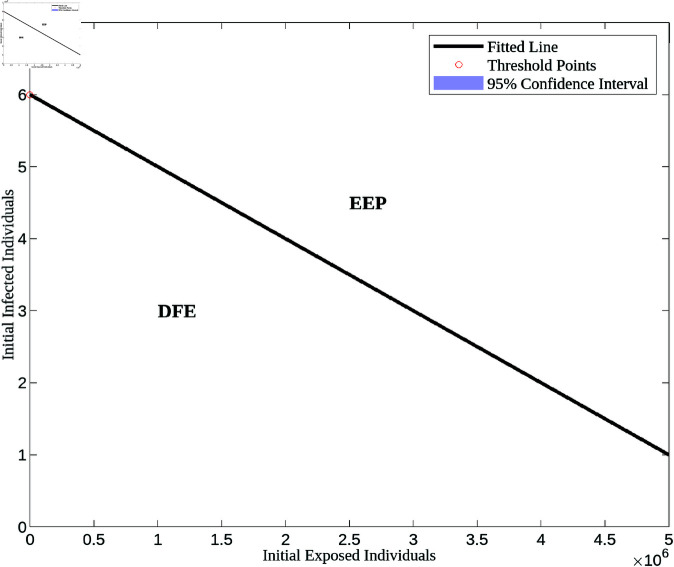
Basin of attraction of DFE and EEP. Figure demonstrating the basin of attraction for DFE and EEP. Here, we set β=2.80026e − 9,*p*  =  3.5, while all other parameters were taken from Table 2. The choice of *p* = 3.5 provides a clearer visualization of both basins. Reducing *p* results in a smaller basin for the EEP, which eventually disappears when p≤1.

As discussed earlier, the outcome of the system—whether it converges to the disease-free equilibrium (DFE) or the endemic equilibrium (EEP)—depends not only on parameter values but also on the initial conditions. In this subsection, we aim to determine the basin of attraction for both equilibria, as illustrated in Fig 7.

Given that our model operates in a five-dimensional state space, we selected *E* and *I* as the key variables for visualizing the basin of attraction for DFE and EEP, as they play a crucial role in disease transmission. To generate this figure, we solved the system of Eq (1) numerically using MATLAB’s ode45 solver [40], systematically varying *E*(0) and *I*(0) in the *EI*-plane while keeping the initial conditions for the other variables constant. The minimum values of *E*(0) and *I*(0) that led to the endemic state were then identified. Using these threshold points, we applied MATLAB’s polyfit function to construct a critical threshold curve, distinguishing the regions where the system converges to either DFE or EEP.

To ensure a complete depiction of the long-term dynamics, we solved the model over a period of 500 years. While this time span may seem quite long, it was necessary to allow the full development of the system’s dynamics (as demonstrated in Fig 6) and to clearly delineate both basins of attraction.

Fig 7 illustrates that if the initial condition falls within the basin of attraction of the disease-free equilibrium, the system remains disease-free. Conversely, if the initial condition lies within the basin of attraction of the endemic equilibrium, the disease persists over time. These results offer a deeper understanding of disease dynamics in the presence of a backward bifurcation induced by exogenous reinfection. Notably, backward bifurcation presents significant challenges in controlling epidemic diseases because it allows the disease to persist even when the basic reproduction number (*R*_0_) is below 1. Unlike in standard models, where reducing *R*_0_ below 1 ensures disease elimination, backward bifurcation introduces multiple stable equilibria, meaning that the epidemic can still persist depending on the initial number of infected individuals. This makes early intervention critical, as a delay in implementing control measures can push the system into the basin of attraction of the endemic equilibrium, making eradication much more difficult.

Moreover, the extended time required for the dynamics to fully develop suggests that the disease may persist for a prolonged period, presenting significant challenges to long-term eradication efforts. In this context, more aggressive interventions are needed to move the system away from the endemic equilibrium. These include increasing vaccination coverage, improving treatment strategies, and implementing stricter contact reduction measures. Even if control measures effectively reduce the disease in certain areas, small changes—like migration, shifts in human behavior, or waning immunity—can cause the disease to re-emerge. This requires ongoing monitoring and long-term intervention strategies.

Having understood this, we derive the type reproduction number in Sect 4, which accounts for both primary transmission and secondary transmission due to exogenous reinfection, offering a comprehensive measure of disease spread. This metric serves as an analogue to the traditional herd immunity threshold, providing critical insights for designing effective non-pharmaceutical intervention strategies. Consequently, an optimal control model is introduced in Sect 5.

## 4 Herd immunity threshold

From the analysis above, we determined that in addition to *R*_0_, the size of the initially exposed and infected populations significantly influences the model’s outcome in the presence of exogenous re-infection. To accurately determine the herd immunity threshold, it is crucial to have a comprehensive understanding of the disease dynamics and all transmission pathways. The basic reproduction number only indicates the total number of secondary infections caused by an infected individual, without accounting for the specifics of multiple transmission routes, if they exist. In contrast, the type reproduction number quantifies the contributions of different transmission routes—whether direct or indirect—to the overall spread of the infection. This measure facilitates an analysis of how various transmission pathways contribute to the disease’s spread, enabling the effective implementation of control strategies.

Several studies have employed the concept of the type reproduction number to investigate disease dynamics. For instance, Islam *et al*. [36] modeled disease transmission through two routes but assumed that both transmissions originated from the susceptible class. Nuraini *et al*. [41] analyzed the type reproduction number by examining both direct human-to-human contact and indirect transmission through vectors. Previous investigations focused on the spread of disease from the susceptible class despite the existence of distinct transmission pathways. In contrast, our model adopts different assumptions; we consider two potential modes of transmission, where forward infection occurs from the susceptible class and backward infection due to exogenous reinfection arises from the recovery class. However, we can directly determine the type reproduction number (*T*_*H*_) because our model includes two reproduction numbers: the basic reproduction number (*R*_0_), representing forward transmission, and the basic reinfection number (*R*_*p*_), which accounts for transmission caused by reinfected individuals from the recovered class. By adding these two reproduction numbers, we obtain the type reproduction number (*T*_*H*_) for our model, given by the equation $T_{H}=R_{0}+R_{p}$.

Additionally, by utilizing the methods and notation from Roberts and Heesterbeek (2003) [42], we can determine the type reproduction number, *T*_*H*_, in our model. Based on their approach, the type reproduction number is expressed as:


$$T_{H}=e^{\prime }K(I-(I-P)K{)}^{-1}e,$$

where *I* is the 2 × 2 identity matrix, *P* is the projection matrix, defined by $P_{11}=1,P_{ij}=0$ when i≠1 or j≠1 and *e* is a unit column vector. Next, we construct the 2 × 2 *K* matrix, where *K*=[*K*_*ij*_]= represents the expected number of secondary infections in class *i* caused by a single infected individual in class *j*. In our model, we assume that the compartments *E* and *I* are infectious. Therefore, the next generation matrix, or *K*-matrix, given by

$$K=[\begin{array}{cc} K_{11} & K_{12}\\ K_{21} & K_{22} \end{array}]$$ (17)

Based on the definition of the next-generation matrix, we have *K*_11_ = *K*_22_ = 0, and *K*_21_ represents the expected number of secondary infections in compartment *I* caused by individuals in *E*, which is given by $K_{21}=\frac{\omega }{\gamma +\delta +\psi }$. And,

K12=Secondary infection arising in compartment Edue to contact of SwithI+exogenous reinfection due to contact of R with I=βS0+pγω+ψ (18)

Thus, the next-generation matrix *K* becomes:

$$K=[\begin{array}{cc} 0 & \frac{\beta S_{0}+p\gamma }{\omega +\psi }\\ \frac{\omega }{\gamma +\delta +\psi } & 0 \end{array}]$$ (19)

After some calculations, the type reproduction number *T*_*H*_ is found to be


$$T_{H}=\frac{\beta \Lambda \omega }{\psi (\omega +\psi )(\gamma +\delta +\psi )}+\frac{p\omega \gamma }{\left(\omega +\psi )(\gamma +\delta +\psi \right)}$$

Thus, the type reproduction number *T*_*H*_ is simply the sum of the basic reproduction number *R*_0_ and the basic reinfection number *R*_*p*_. It reflects both forward transmission from the susceptible class and backward transmission from the recovered class, capturing the contributions of both pathways. This dual perspective makes it easier to interpret and communicate the dynamics of infection spread. Below, we derive the stability requirements for our model in terms of the type reproduction number.

i) When *T*_*H*_<1, the disease will eventually die out.
ii) When *T*_*H*_>1 with *R*_0_>1, the disease persists, regardless of the value of *R*_*p*_.
ii) However, when *T*_*H*_>1 with *R*_*p*_>1 and $R_{0}\in (R_{c},1)$, both the disease-free state and the endemic state coexist.

**Fig 8:**
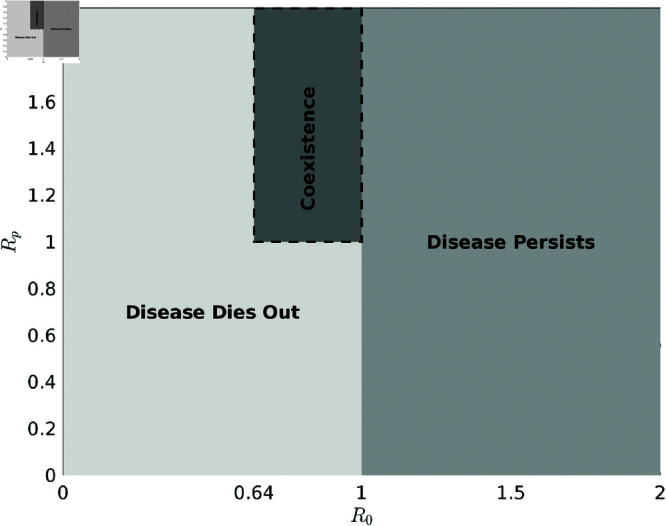
Phase portrait showing outcomes based on *T*_*H*_. Phase portrait of disease dynamics illustrating the relationship between *R*_0_ and *R*_*p*_ based on $T_{H}=R_{0}$  +  *R*_*p*_ values, indicating regions of disease extinction, persistence, and coexistence.

The phase diagram of disease dynamics shown in Fig 8 illustrates the relationship between *R*_0_ and *R*_*p*_ based on $T_{H}=R_{0}+R_{p}$ values, highlighting the regions where the disease can die out, persist, or coexist. From the above analysis, it is evident that while *R*_0_ and *R*_*p*_ provide valuable insights into disease transmission, they offer only a partial view of the overall dynamics. They mainly focus on specific aspects of the epidemic, such as the initial spread and the potential for re-infection. In contrast, *T*_*H*_ gives a more complete understanding by accounting for multiple transmission pathways. It offers a holistic perspective on whether the disease is likely to fade out or continue circulating in the population. If strategies are implemented to keep *T*_*H*_ below 1, the disease will eventually die out. However, if *T*_*H*_ exceeds 1 due to any of the transmission routes, the disease will persist and continue to spread in the community.

## 5 Interventions

Herd immunity occurs when a significant portion of the population gains immunity to a specific infectious disease, either through vaccination or prior infections. This helps reduce the overall spread of the disease and protects those who are not immune, such as individuals who cannot be vaccinated for medical reasons or those with weakened immune systems. However, where vaccination is not available or equally accessible, another way to achieve this is by ensuring a significant portion of the population remains protected from infection.

In our model, we consider the case of reinfection, indicating that natural immunity alone cannot provide lasting protection. As a result, classical herd immunity cannot be achieved in the traditional sense. For this reason, we examine herd immunity as a reduction in HPV prevalence achieved through non-pharmaceutical interventions.

To quantify the impact of both transmission routes, we previously introduced the type reproduction number (*T*_*H*_). The herd immunity threshold based on *T*_*H*_ is given by V≥1 − $\frac{1}{T_{H}}=1$ − $\frac{1}{R_{0}+R_{p}}.$ Since our model does not account for vaccination and includes exogenous reinfection, the traditional concept of herd immunity is not directly applicable. However, the quantity *V* suggests that if a fraction *V* of the population can be protected through non-pharmaceutical interventions, the disease will eventually be eradicated. This indirect form of herd immunity establishes mathematical rigor, highlighting that effective control strategies, in the absence of vaccination, must mitigate both primary transmission and secondary transmission driven by exogenous reinfection.

Designing an optimal control model to mitigate HPV transmission through non-pharmaceutical interventions requires a comprehensive assessment of key socio-economic infrastructure factors. In the context of Bangladesh, controlling the transmission of HPV presents significant challenges, particularly in rural areas where access to education and healthcare is limited. Cultural taboos and a lack of awareness about sexual health leave many women, especially in these regions, unaware of the risks associated with HPV and cervical cancer. Even when information is available, financial constraints often prevent individuals from accessing preventive measures. Furthermore, in these communities, women frequently have limited autonomy in sexual health decisions, with many being unable to negotiate safe practices, thereby increasing their vulnerability to HPV infection.

In contrast, in urban areas, sex workers live in isolated communities with limited access to sexual health services and public health education. These communities are often invisible to the broader public, and the lack of awareness and resources makes them especially vulnerable to HPV transmission. Despite efforts to control the spread of HPV, these hidden, underserved populations continue to drive the virus’s spread.

Below, we present an optimal control model that incorporates a control effort 0≤u(t)≤1 representing the adherence to safe sex practices. The value is constrained to be less than 1 because, despite rigorous public education campaigns, not all individuals adhere to safe sex guidelines. This control effort will reduce the transmission rate, as given by the expression βη(1 − u(t))IS. Here, the parameter 0≤η≤1 quantifies the reduction in transmission due to the implementation of safe sex practices. The value of η∈[0,1] represents the effectiveness of the preventive measures: when η=0, the measures completely halt transmission, whereas η=1 indicates that the measures have minimal impact on transmission.

As previously discussed in Sects 3.5.1 and 5, the exogenous reinfection-driven backward bifurcation introduces significant challenges to controlling disease transmission. We have observed that while reducing the primary transmission can lower the basic reproduction number (*R*_0_) below 1, the disease may still persist. Consequently, control strategies such as *u*(*t*), which measure adherence to safe sex practices, may not be sufficient on their own to halt transmission. In this context, it is reasonable to assume that individuals initially infected due to unsafe sexual practices, particularly within the sociocultural context of Bangladesh as discussed earlier in this section, may remain vulnerable to re-engaging in such behaviors after recovery, further contributing to the spread of the disease. Therefore, targeting education efforts for recovered individuals, possibly through hospitals, public media, or community-based initiatives, can play a crucial role in curbing backward transmission pathways. By promoting awareness and encouraging safe sex practices, these efforts could significantly reduce the impact of reinfection. This approach of community education could be effectively implemented through the Directorate General of Health Services (DGHS) in Bangladesh.

Therefore, we redefine the reinfection term pβRI in model (1) as $f(R,I)=\frac{p\beta RI}{1+\alpha (t)I}$. The term 1  +  α(t)I primarily represents the saturation effect on reinfection, which intensifies as *I* increases. The parameter α(t) captures the rate at which this saturation effect is triggered, reflecting the time-dependent awareness fostered through community education. Notably, α(t) is assumed to increase with *I*, reflecting heightened awareness as the infection rate rises. For simplicity, we approximate α(t) as a linear function of *I*, i.e., $\alpha (t)=\alpha_{0}$  +  $\alpha_{1}I$, where $\alpha_{0}=0$. To streamline notation, we use α in place of $\alpha_{1}$, leading to the following optimal control model.

$$\frac{dS}{dt}=\Lambda -\beta \eta (1-u(t))IS-\psi S\;\frac{dE}{dt}=\beta \eta (1-u(t))IS+\frac{p\beta RI}{1+\alpha I^{2}}-\psi E-\omega E\;\frac{dI}{dt}=\omega E-\gamma I-\delta I-\psi I\;\frac{dR}{dt}=\gamma I-\frac{p\beta RI}{1+\alpha I^{2}}-\psi R\;\frac{dC}{dt}=\delta I-\mu C-\psi C$$ (20)

**Fig 9:**
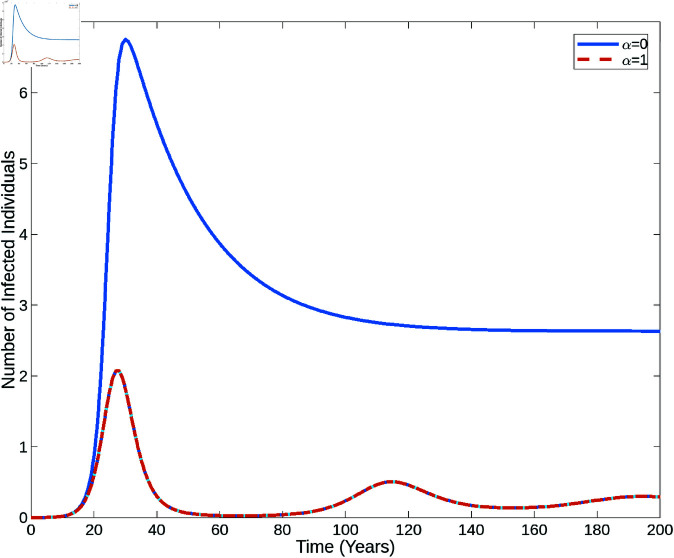
Positive effect of community education. Figure showing the dynamics without preventive measure *u*(*t*), corresponding to α=0 and α=1.

When α=0, the system reverts to the original system. The effect of α is demonstrated in Fig 9. This figure clearly illustrates that community education plays a crucial role in significantly reducing the number of infected individuals. The oscillatory nature of the solution holds profound biological significance. When infection levels rise substantially, individuals tend to adhere more strictly to community guidelines on safe sex practices, leading to a marked decline in new cases. However, as the perceived threat diminishes, complacency sets in, causing a relaxation of these precautions, which in turn triggers a resurgence in infections.

Having thoroughly examined the impact of community education, we now introduce the optimal control model. Our objective is to mitigate the spread of HPV and reduce cervical cancer cases. To achieve this, we seek to minimize the following functional

$$J(u(t))=\int_{0}^{T}\left(\frac{A}{2}u^{2}(t)+B_{1}I(t)+B_{2}C(t)\right)\;dt$$ (21)

The constants *B*_1_ and *B*_2_ represent the per capita costs associated with infection and cervical cancer, respectively, while *A* denotes the cost associated with implementing the control measures *u*. This can be achieved mathematically by determining the optimal controls ${\text{u}}^{*}$ so that minimize the cost functional (21).

The Hamiltonian function H has the following form:

$$H=\frac{A}{2}u^{2}+B_{1}I+B_{2}C\;\;+\lambda_{1}\left[\Lambda -\beta \eta (1-u(t))IS-\psi S\right]\;\;+\lambda_{2}\left[\beta \eta (1-u(t))IS+\frac{p\beta RI}{1+\alpha I^{2}}-\psi E-\omega E\right]\;\;+\lambda_{3}\left[\omega E-\gamma I-\delta I-\psi I\right]+\lambda_{4}\left[\gamma I-\frac{p\beta RI}{1+\alpha I^{2}}-\psi R\right]\;\;+\lambda_{5}\left[\delta I-\mu C-\psi C\right]$$ (22)

The adjoint variables $\lambda_{1}$, $\lambda_{2}$, $\lambda_{3}$, $\lambda_{4}$, and $\lambda_{5}$, corresponding to the Hamiltonian function of the system, are obtained by solving the following adjoint system


$$\lambda_{1}^{\prime }=\beta \eta (\lambda_{1}-\lambda_{2})(1-u(t))I+\psi \lambda_{1},\;\lambda_{2}^{\prime }=\omega (\lambda_{2}-\lambda_{3})+\psi \lambda_{2},\;\lambda_{3}^{\prime }=\beta \eta (1-u(t))S(\lambda_{1}-\lambda_{2})+\frac{p\beta R(1-\alpha I^{2})(\lambda_{4}-\lambda_{2})}{(1+\alpha I^{2}{)}^{2}},\;\;+\lambda_{3}(\gamma +\delta +\psi )-\lambda_{4}\gamma -\lambda_{5}\delta -B_{1},\;\lambda_{4}^{\prime }=\frac{p\beta I(\lambda_{4}-\lambda_{2})}{\left(1+\alpha I^{2}\right)}+\psi \lambda_{4},\;\lambda_{5}^{\prime }=\lambda_{5}(\psi +\mu )-B_{2},$$

with the transversality conditions $\lambda_{i}(T)$=0; i=1,2,3,4,5. The required optimal control ${\text{u}}^{*}(\text{t})$ is stated by the following expressions:

$$u^{*}(t)=\min\left(1,\max\left(0,\frac{(\lambda_{2}^{*}(t)-\lambda_{1}^{*}(t))\beta I^{*}S^{*}}{A}\right)\right)$$ (23)

**Fig 10:**
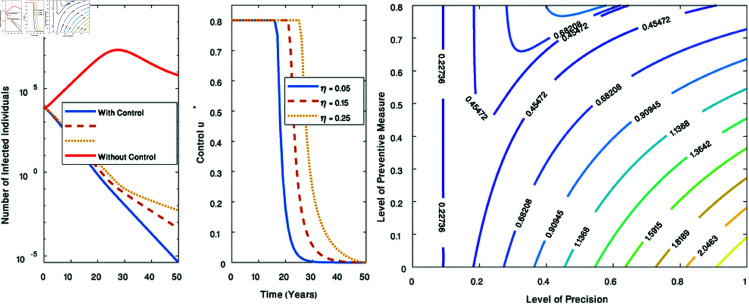
Key results of optimal control model. The figure on the left illustrates the disease dynamics when preventive measures are implemented with varying levels of precision, while the figure on the right depicts the dynamics under different intervention scenarios. In both cases, the optimal control model was simulated over the time span t∈[0,50].

The existence of optimal control [43] is discussed in S3 Appendix. We apply the Forward–Backward Sweep method to numerically solve the optimal control system [33]. The key results of the optimal control model, simulated over the time span t∈[0,50], are shown in Fig 10. The graph on the left illustrates the dynamics of the disease under preventive measures with varying levels of precision (η). It shows that as η increases, the duration required for the intervention to effectively control the disease also increases. The rationale for selecting this time span is that it likely represents the maximum feasible duration for devising and executing an intervention strategy to eradicate the disease from society.

Further simulations, shown on the right side of Fig 10, detail the dynamics under various intervention scenarios. The results indicate that the disease remains largely controlled when the precision level of preventive measures is consistently high across all levels of effective intervention. However, when the precision of preventive measures is poor, the disease persists and remains endemic by the end of the simulation period. In this scenario, inadequate precision leads to a surge in new infections, which, in turn, amplifies the effects of exogenous reinfection. The increased influx of new cases fuels reinfection, prolonging the time required to bring the disease under control.

**Fig 11:**
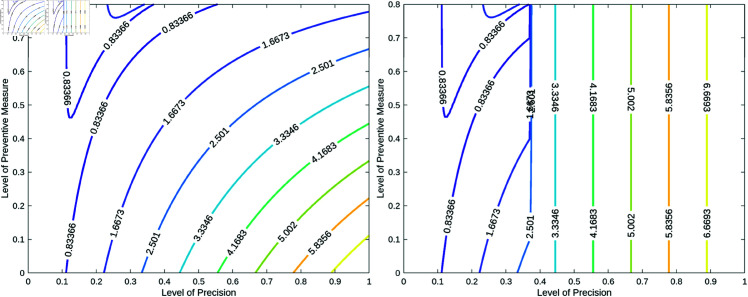
Dynamics corresponding to a high transmission rate. Figures illustrating the detailed disease dynamics under a high transmission rate. The left figure depicts the controlled reproduction number when α=1, while the right figure shows the controlled reproduction number for α=0.

To gain a deeper understanding of the challenges in controlling the disease caused by reinfection, we re-simulate the optimal control model under a relatively high transmission rate (β). In this scenario, we use twice the fitted value of β, resulting in a reproduction number of approximately 5, consistent with values reported in previous studies [39]. It is important to note that we simulate the model for both α=0 and α=1 over an extended time span, t∈[0,200]. Although this duration is relatively long, it serves an experimental purpose—allowing us to investigate the role of reinfection in driving the worst-case scenario of disease dynamics. The results are shown in Fig 11.

The figure on the left shows that when α=1, the controlled reproduction number varies non-linearly with both the intervention effort (*u*) and the precision level (η), offering greater flexibility in controlling the disease even at lower precision levels. This suggests that with a high intervention effort, relatively imprecise measures can still effectively reduce the disease burden. In contrast, when α=0, as shown on the right of Fig 11, the non-linear relationship disappears as the precision level decreases, and the disease can only be controlled at very high precision levels, regardless of the intervention effort. In this case, the intervention’s effectiveness becomes heavily reliant on achieving high precision, highlighting the critical need for fine-tuning the intervention strategy to optimize outcomes.

Overall, the relationship between the intervention effort (*u*) and the precision level (η) for different values of α reveals a clear trend: increasing η typically requires a higher intervention effort to sustain similar levels of disease control. This is especially noticeable when community education is present, as in the case where α=1. Here, even smaller values of η allow for significant control over disease dynamics, whereas larger values of η necessitate a proportional increase in intervention effort to offset the reduced precision. However, this phenomenon disappears when community education is absent, that is, when α=0, meaning reinfection exerts its full impact on the disease dynamics.

## 6 Conclusion

This study examines the critical role of HPV transmission dynamics and the challenges of exogenous reinfection, particularly in the absence of vaccination. By developing a deterministic model that incorporates reinfection, we offer valuable insights into the complexity of HPV spread. The observation of backward bifurcation underscores the need for a multifaceted control strategy that targets both primary and secondary infections. Our analysis reveals that addressing only primary infections is inadequate; effective eradication requires tackling exogenous reinfections as well.

The optimal control solutions presented in this study offer a promising strategy for countries like Bangladesh, where limited vaccine access poses a challenge, and community-driven education on safe sexual practices remains essential yet evolving. The findings indicate that targeted preventive measures, coupled with comprehensive community education, can significantly mitigate the health risks associated with HPV. Implementing these strategies could play a crucial role in reducing the burden of cervical cancer and improving public health outcomes. Furthermore, our results emphasize the importance of refining intervention strategies to maximize effectiveness, as their success depends heavily on the precision of implementation. Insufficient precision, on the other hand, leads to delayed control of disease transmission, triggering a surge in new infections that subsequently intensifies exogenous reinfection. This cycle not only accelerates disease transmission, but also prolongs the time needed to achieve meaningful control, underscoring the urgency of well-calibrated interventions.

The validation of our model using real-world data from Bangladesh highlights its relevance in understanding HPV dynamics. However, reliance on VIA for screening introduces inherent limitations in sensitivity and specificity, potentially skewing cervical cancer and HPV prevalence estimates. Additional biases may arise from underreporting, incomplete rural coverage, and variability in screening quality. Despite these challenges, the dataset remains one of the most comprehensive sources for estimating cervical cancer incidence and HPV infection rates in Bangladesh, providing valuable insights for model calibration. While disease dynamics may differ across datasets, requiring tailored intervention strategies, this study serves as a critical foundation for further exploration and refinement in this direction.

In summary, the results indicate that a well-structured, high-efficacy intervention plan plays a crucial role in controlling disease transmission. However, imprecise implementation may lead to delays, increasing the risk of reinfection and ultimately prolonging disease persistence, potentially resulting in intervention failure. In addition, migration may further complicate disease control efforts, although analysis of its impact is beyond the scope of this study. Future research could develop a more sophisticated model that accounts for migration and the complexities introduced by exogenous reinfection, providing deeper insights into whether the nonpharmaceutical intervention strategies examined here are sufficiently effective or if vaccination remains the definitive solution for alleviating the HPV-related burden, as explored in [14,39]. Furthermore, an extension of this study could focus on communities where partial vaccination is available, offering a valuable framework to assess whether the strict precision required for implementing the proposed interventions may be eased while still effectively controlling and containing the spread of HPV.

## Supporting information

S1 Appendix Global stability analysis of disease-free equilibrium. (PDF)

S2 Appendix Backward bifurcation threshold. (PDF)

S3 Appendix Existence of optimal control. (PDF)

S1 Table VIA test results data. (PDF)
