# Supplementary material for: Herd immunity and prevention in HPV transmission with exogenous reinfection
Source: PLoS One. 2025 Jul 11;20(7):e0327233. doi: 10.1371/journal.pone.0327233 (PMC12250543; doi:10.1371/journal.pone.0327233)
Supplement: S1 Appendix — (PDF) [file pone.0327233.s001.pdf]

S1 Appendix. Global stability analysis of disease-free equilibrium.

Theorem 1. *The disease-free equilibrium E_0 is globally asymptotically stable when $T_H < 1$, otherwise it is unstable.*

Proof. We consider the following Lyapunov candidate function

$$V = k_1 E + k_2 I + k_3 R,$$

where $k_1 = \dfrac{\omega}{(\omega + \psi)(\gamma + \delta + \psi)}$, $k_2 = \dfrac{1}{(\gamma + \delta + \psi)}$, and $k_3 = \dfrac{p\omega}{(\omega + \psi)(\gamma + \delta + \psi)}$.

After some simplifications and manipulations, we obtain

$$\frac{dV}{dt} \approx (T_H - 1)I - \frac{p\omega\psi R}{(\omega + \psi)(\gamma + \delta + \psi)}.$$

Therefore, $\frac{dV}{dt} < 0$ when $T_H < 1$, suggesting that the DFE is globally asymptotically stable when $T_H < 1$. □
