# Supplementary material for: Herd immunity and prevention in HPV transmission with exogenous reinfection
Source: PLoS One. 2025 Jul 11;20(7):e0327233. doi: 10.1371/journal.pone.0327233 (PMC12250543; doi:10.1371/journal.pone.0327233)
Supplement: S2 Appendix — (PDF) [file pone.0327233.s002.pdf]

S2 Appendix. Backward bifurcation threshold.

To determine the backward bifurcation threshold R_c, we apply the discriminant from equation (9) which is $\Delta = B^2 - 4AC$. By setting $\Delta = 0$ and rewriting the equation in terms of β, we can determine the critical value of the transmission coefficient denoted by β_c. The discriminant is equal to zero for β_c, which is the critical value of β. After some calculation, we obtain the equation,

$$a\beta_c^2 + b\beta_c + c = 0$$

where,

$$\begin{aligned}
a &= p^2\omega^2\Lambda^2 \\
b &= 2p^2\omega^2\Lambda\gamma\psi + 4p\Lambda\omega\psi(\omega+\psi)(\delta+\psi+\gamma) \\
&\quad - 4p\gamma\psi\Lambda\omega^2 - 2p\omega\Lambda\psi(\omega+\psi)(\delta+\psi+\gamma) - 2p^2\omega\Lambda\psi(\omega+\psi)(\delta+\psi+\gamma) \\
c &= \omega^2p^2\gamma^2\psi^2 - 2p\psi^2(\omega+\psi)^2(\delta+\psi+\gamma)^2 \\
&\quad + \psi^2(\omega+\psi)^2(\delta+\psi+\gamma)^2 + p^2\psi^2(\omega+\psi)^2(\delta+\psi+\gamma)^2 \\
&\quad + 4p\omega\gamma\psi^2(\omega+\psi)(\delta+\psi+\gamma) - 2\omega p^2\gamma\psi^2(\omega+\psi)(\delta+\psi+\gamma) \\
&\quad - 2\omega p\gamma\psi^2(\omega+\psi)(\delta+\psi+\gamma)
\end{aligned}$$

Now the critical transmission rate,

$$\beta_c = \frac{-b \pm \sqrt{b^2 - 4ac}}{2a}$$

Therefore the value of the backward bifurcation threshold is obtained by replacing parameter β in R_0 with β_c which yields

$$R_c = \frac{\sqrt{b^2 - 4ac} - b}{2p^2\omega\Lambda\psi(\omega+\psi)(\gamma+\delta+\psi)}$$

Here, R_c represents the threshold for backward bifurcation, which occurs in the range $R_0 \in (0,1)$ when $R_p > 1$, while R_p corresponds to the basic reinfection number. R_p serves as the threshold that separates backward from forward bifurcation: when $R_p > 1$, backward bifurcation takes place, whereas forward bifurcation occurs when $R_p \leq 1$. The interval $(R_c, 1)$ defines the region where backward bifurcation occurs. Specifically, for every $R_0 \in (R_c, 1)$, the system will undergo backward bifurcation when $R_p > 1$.

As R_p increases beyond 1, the value of R_c decreases, thereby expanding the bifurcation region $(R_c, 1)$. In contrast, when R_p decreases, R_c increases and approaches 1, causing the backward bifurcation region to shrink and eventually disappear when $R_p = 1$.
