# Supplementary material for: Herd immunity and prevention in HPV transmission with exogenous reinfection
Source: PLoS One. 2025 Jul 11;20(7):e0327233. doi: 10.1371/journal.pone.0327233 (PMC12250543; doi:10.1371/journal.pone.0327233)
Supplement: S3 Appendix — (PDF) [file pone.0327233.s003.pdf]

S3 Appendix. Existence of Optimal Control.

The control is bounded by $0 \leq u(t) \leq 1$. Therefore, the transmission term $\beta\eta(1-u(t))IS$ is also bounded and finite, implying that the state space is bounded in the positive orthant. This suggests that both the control set and the state space are non-empty. The objective function (21) consists of two parts: one quadratic in $u(t)$ and the other linear in I and C. Thus, the objective function is convex.

Lastly, we choose $c_1 = A$ and $\frac{c_2}{2} = min\{B_1 I(t), B_2 C(t)\}$ and $\rho = 2$, then the integrand satisfies the following inequality

$$\frac{A}{2}u^2(t) + B_1 I(t) + B_2 C(t) \geq c_2 + c_1|u|^2$$

which ensures that the objective function has a lower bound. This lower bound guarantees that the integrand is strictly positive, meaning the total cost functional is finite. Therefore, this ensures the existence of an optimal control [1].

References

[1] Islam MH, Masud MA. Investigation of Rabies Control in Free-roaming Dogs: A Mathematical Modelling Approach From Bangladesh. In: One Health for Dog-mediated Rabies Elimination in Asia: A Collection of Local Experiences. CABI GB; 2023. p. 85–98.
