# Supplementary material for: Herd immunity and prevention in HPV transmission with exogenous reinfection
Source: PLoS One. 2025 Jul 11;20(7):e0327233. doi: 10.1371/journal.pone.0327233 (PMC12250543; doi:10.1371/journal.pone.0327233)
Supplement: S1 Table — (PDF) [file pone.0327233.s004.pdf]

S1 Table. VIA test results data. The following table contains the VIA test results data of Bangladesh [1].

Table 1: **VIA Positive (2019-2023).**

**YEAR**	**VIA Positive**
2019	7502
2020	6899
2021	12187
2022	16039
2023	21963

References

[1] National Cervical Cancer Screening and Treatment Program Dashboard; 2024. `https://nccbcst.bsmmu.ac.bd/dashboard` (Date accessed: 30.09.2024).
